# Preparation and Characterization of Silymarin-Conjugated Gold Nanoparticles with Enhanced Anti-Fibrotic Therapeutic Effects against Hepatic Fibrosis in Rats: Role of MicroRNAs as Molecular Targets

**DOI:** 10.3390/biomedicines9121767

**Published:** 2021-11-25

**Authors:** Abdullah Saad Abdullah, Ibrahim El Tantawy El Sayed, Abdel Moneim A. El-Torgoman, Noweir Ahmad Alghamdi, Sami Ullah, S. Wageh, Maher A. Kamel

**Affiliations:** 1 Department of Chemistry, Faculty of Science, Menoufia University, Shebin El Koom 13829, Egypt; ibrahimtantawy@science.menofia.edu.eg (I.E.T.E.S.); abdelmoneam.abdelkader@science.menofia.ed (A.M.A.E.-T.); 2 Department of Physics, Faculty of Science, Albaha University, Alaqiq 65779, Saudi Arabia; naa.alghamdi@bu.edu.sa; 3 Research Center for Advanced Materials Science (RCAMS), King Khalid University, P.O. Box 9004, Abha, 61413, Saudi Arabia; samiali@kku.edu.sa; 4 Department of Chemistry, College of Science, King Khalid University, P.O. Box 9004, Abha 61413, Saudi Arabia; 5 Department of Physics, Faculty of Science, King Abdulaziz University, Jeddah 21589, Saudi Arabia; 6 Physics and Engineering Mathematics Department, Faculty of Electronic Engineering, Menoufia University, Menoufia 32952, Egypt; 7 Department of Biochemistry, Medical Research Institute, Alexandria University, Alexandria 21516, Egypt

**Keywords:** liver fibrosis, silymarin, gold nanoparticles, epigenetics, microRNAs, oxidative stress

## Abstract

Background: The main obstacles of silymarin (SIL) application in liver diseases are its low bioavailability, elevated metabolism, rapid excretion in bile and urine, and inefficient intestinal resorption. The study aimed to synthesize and characterize silymarin-conjugated gold nanoparticles (SGNPs) formulation to improve SIL bioavailability and release for potentiating its antifibrotic action. Methods: Both SGNPs and gold nanoparticles (GNPs) were prepared and characterized using standard characterization techniques. The improved formulation was assessed for in vitro drug release study and in vivo study on rats using CCl_4_ induced hepatic fibrosis model. SIL, SGNPs, and GNPs were administered by oral gavage daily for 30 days. At the end of the study, rats underwent anesthesia and were sacrificed, serum samples were collected for biochemical analysis. Liver tissues were collected to measure the genes and microRNAs (miRNAs) expressions. Also, histopathological and immunohistochemistry (IHC) examinations of hepatic tissues supported these results. Results: The successful formation and conjugation of SGNPs were confirmed by measurements methods. The synthesized nanohybrid SGNPs showed significant antifibrotic therapeutic action against CCl_4_-induced hepatic damage in rats, and preserved normal body weight, liver weight, liver index values, retained normal hepatic functions, lowered inflammatory markers, declined lipid peroxidation, and activated the antioxidant pathway nuclear factor erythroid-2-related factor 2 (NRF2). The antifibrotic activities of SGNPs mediated through enhancing the hepatic expression of the protective miRNAs; miR-22, miR-29c, and miR-219a which results in suppressed expression of the main fibrosis mediators; TGFβR1, COL3A1, and TGFβR2, respectively. The histopathology and IHC analysis confirmed the anti-fibrotic effects of SGNPs. Conclusions: The successful synthesis of SGNPs with sizes ranging from 16 up to 20 nm and entrapment efficiency and loading capacity 96% and 38.69%, respectively. In vivo studies revealed that the obtained nano-formulation of SIL boosted its anti-fibrotic effects.

## 1. Introduction

Despite significant scientific advances in hepatology, the number of people suffering from liver diseases is increased in recent years, and the death rate remains high [1]. In 2017, an estimated 1.5 billion people globally have chronic liver disease (CLD), and the age-standardized incidence of CLD and cirrhosis is 20.7/100,000, a 13% rise since 2000 [2,3]. Every year, nearly 2 million people die as a result of liver disease; 1 million as a result of cirrhosis complications, and 1 million as a result of viral hepatitis and hepatocellular carcinoma (HCC) [4].

Liver fibrosis is a response generated as a result of sustained and chronic liver injury stimulated by many factors such as hepatitis viral disease, immune system hepatitis, alcohol consumption, non-alcoholic steatohepatitis (NASH), non-alcoholic fatty liver disease (NAFLD), and chemically toxic substances like carbon tetrachloride (CCl_4_) [5]. On a cellular level, Liver fibrosis leads to the development of liver cirrhosis and HCC at the end-stage [6]. Liver fibrosis is distinguished by the excessive accumulation of extracellular matrix (ECM) proteins, such as collagen and fibronectin, produced by activated hepatic stellate cells (HSCs) [7]; this leads to hepatic architecture distortion and function dysfunction and is thus a prominent characteristic of a cirrhotic liver. The quiescent HSCs with gradual chronic inflammation convert into myofibroblast-like cells, which are characterized by the appearance of cytoskeleton protein α smooth muscle actin (α-SMA) and collagen considered as a biomarker for HSCs activation. Transforming growth factor-beta 1 (TGF-β1) is the most effective activator of HSCs and converts it from static HSCs to the phenotype of myofibroblast to express α-SMA [8,9]. Furthermore, it was reported that an interrelationship between the nuclear factor erythroid-2-related factor 2 (Nrf2) and TGF-β1 pathways promote the development of HCC [10]. Previous studies have shown that Nrf2 adversely acts against fibrotic TGF-β1 signaling [11] and TGF-β1 stimulates the generation of reactive oxygen species production (ROS) by inhibiting Nrf2 [12].

MicroRNAs (miRNAs) are short non-coding RNAs (20–22 nucleotides) that serve as posttranscriptional inhibitors of gene expression by binding to their target mRNAs’ incomplete complementary sequences at the 3′ untranslated region (3′ UTR) and inducing mRNA degradation or translational repression [13]. MiRNAs play an important role in regulating liver fibrogenesis by controlling the expression of multiple signaling components, transcription factors, and cofactors [14]. MiR-22 [15], miR-29c [16], and miR-219a [17] have been shown to target various genes during liver fibrosis. TGFβR1 [18], COI3A1 [19], and TGFβR2 [20] genes are three of the genes that have been predicted as potential targets for miRNAs and are primarily involved in fibrogenesis.

Silymarin (SIL) is a flavonolignans mixture extracted from the milk thistle (*Silybum marianum* (L.) Gaertn) seeds. Silibinin is the most active component in this extract (silybin A and silybin B in a 50:50 ratio); the remaining components are silydianin, silycristin, isosilybin A, isosilybin B, isosilycristin, and taxifolin [21]. SIL is an important hepatoprotective substance used in clinical applications, known for its antioxidant and anti-inflammatory properties for hepatic disorders particularly liver fibrosis. The main obstacles of SIL application are, its water solubility-related bioavailability (0.5 g/L), elevated metabolism in extensive phase II, rapid excretion in bile and urine, and inefficient intestinal resorption [22,23]. Several attempts to solubilize SIL were made, however, neither of these attempts showed any pharmacological results achievements [24]. The new approach of nanotechnology may play important role in enhancing the bioavailability and therapeutic properties of compounds, in particular, plant compounds [25].

The gold in its bulk form has long been traditionally known to be an inert noble transition metal, with some therapeutic value and even medicinal properties, thus, Gold nanoparticles (GNPs) are often assumed relatively non-cytotoxic as well [26]. GNPs have been deemed the most promising delivery system in drug delivery in the case of metallic nanoformulations because of their small size, high solubility, and chemical inertness in a biological environment, as well as their biocompatibility [26]. The simple syntheses and surface modifications; much greater surface area to volume ratio in GNPs facilitates several a hundred molecules to be absorbed on its surface [27], good enhanced and tunable optical properties, as well as excellent biocompatibility feasible for clinical conditions, have taken them to the frontline of cancer research in recent years [28]. However, there are various reports of the extent of the toxicity of these particles due to their different alterations, surface functional attachments, shape, and size [29]. In this study, we utilized SIL as a reducing agent to produce GNPs from the reduction process in the synthesizing procedure of SGNPs. In reality, phytochemicals such as flavonolignans, flavonoids, polyphenolic compounds, and alkaloids found in SIL serve as reducing and stabilizing agents for the synthesis of SGNPs. The reduction capability of SIL was expected to lead to rapid nucleation of the chloroauric acid reaction [30]. These results have recently been documented in the synthesis of gold nanoparticles using curcumin [31] and cinnamon bark extract [32]. The present work was aimed to prepare and characterize SGNPs to improve the antifibrotic potential of SIL in a rat model of CCl_4_-induced liver fibrosis.

## 2. Materials and Methods

### 2.1. Chemicals and Regent

Tetrachlorauric acid HAuCl_4_.H_2_O was purchased from Electron Microscopy Sciences (Hatfield, PA, USA), Silymarin (SIL) powder was generously gifted by Egyptian Group for Pharmaceutical Industries (Cairo, Egypt), Tri-Sodium Citrate 2- Hydrate PA-ACS (C_6_H_5_Na_3_O_7_.2H_2_O); MW 294.10 was obtained from Panreac Quimica (Barcelona, Spain), Sodium Tripolyphosphate, MW 367.86 was procured from alpha Chemika (Mumbai, India). All other chemicals and reagents used throughout the experiments were of the highest analytical grade available. Ultrapure water (Milli-Q) was used in all syntheses.

### 2.2. Preparation and Characterization of Nanoparticles 

#### 2.2.1. Preparation of Gold Nanoparticles

Gold Nanoparticles (GNPs) were prepared according to the method proposed by McFarland et al. [33]. The GNPs were prepared from the reduction of HAuCl_4_.H_2_O (Au^3+^, HAuCl_4_) to neutral gold (Au^0^) in the presence of a reducing agent Trisodium Citrate Dihydrate (TCD). Briefly, 20 mL of 1.0 mM HAuCl_4_.H_2_O was added to a 50 mL conical flask on a stirring hot plate. To the rapidly stirred boiling solution, 2 mL of a fresh TCD solution was added with simultaneous stirring and stopping heating. The gradual change in the color of the gold chloride solution from yellow into dark blue and then a ruby red color within a few minutes indicates the formation of colloidal GNPs, and the solution was kept at stirring for an additional ~40 min, and then left to cool to room temperature. The solution was centrifuged twice at 14,000 rpm for 30 min and the precipitate was rinsed with water between each centrifugation [34] the supernatant was removed and the precipitate was then re-suspended in the appropriate volume of ultra-pure water. Lastly, the pellet was resuspended in ultra-pure water and sonicated for 15 min using a bath sonicator (Power sonic 405.Korea) and stored at 4 °C and in the dark to minimize the photo-induced oxidation.

#### 2.2.2. Synthesis of Silymarin/Gold Nanoparticles

The biogenic gold nanoparticles were synthesized from SIL for the synthesis of silymarin conjugated gold nanoparticles (SGNPs). This method was adopted by Kabir et al. [35]. SGNPs were synthesized by reducing HAuCl_4_ in presence of SIL at different concentration values. Experiments were performed individually to determine the precise molar ratio of SIL to HAuCL_4_ necessary for the optimal reaction. A constant concentration of ionic gold (1.0 mM/5 mL, 0.1 M stock solution) was reacted with various concentrations of SIL (1.0 mM, 2.0 mM, 2.5 mM/10 mL) at room temperature via a magnetic stir plate in each process. The results were accompanied by the following observations; different timeframes for completion of the reaction 1 h, 40 min, 30 min, respectively. The color change was visually monitored at the beginning of the reaction of pale yellow, light gray, the final colors were obtained at the end of each reaction as follows; dark violet-red, maroon red, burgundy red, respectively. Briefly, 10 mL of different concentrations of SIL (dissolved in 0.1 M of NaOH solution) were freshly prepared daily and shook with a vortex shaker. 5 mL of 1.0 mM HAuCl_4_.H_2_O was prepared and stirred for 5 min. 1 mL of SIL solution was slowly added drop-wise using a glass burette into 5 mL of an aqueous solution HAuCl_4_.H_2_O with continuous stirring on a magnetic stirrer. The mixed solution was stirred vigorously for 30 min at room temperature. The gradual change in the color of the mixed solution was depending upon the different concentrations of SIL. The purification process was adopted from Sindhu et al. [36]. First, the final reaction mixture was kept at room temperature for the next day, and nanoparticles were separated by using a centrifuge at a speed of 10,000 rpm for 15 min in (Histam Plus-Rh Centrifuge 18,000, Spain) at a temperature of 14 °C. The supernatant was removed and stored for further characterization and the product pellet obtained was resuspended twice in Milli Q water and recentrifuged at the same speed to remove any unreacted SIL or HAuCl_4_. The concentrated precipitate was finally diluted in Milli Q water and preserved at 4 °C for further characterizations. For FTIR and in vitro drug release measurements, the isolated SGNPs were lyophilized to obtain dry powder using (Alpha 2–4 LD plus Christ, Germany).

### 2.3. Physicochemical Characterizations of Nanoparticles

#### 2.3.1. UV–Visible Spectrophotometry

UV-visible spectra were recorded between 190 and 1100 nm for confirming the formation and stability of nanoparticles. The samples were filled in a quartz cuvette with a length of 1 cm of light path and the spectra of light absorption were given by reference to Milli Q water. The beak value of the UV-visible was documented.

#### 2.3.2. Dynamic Light Scattering (DLS) and Zeta Potential (ZP) Analysis

The particle size (hydrodynamic diameter), polydispersity index (PDI), and ZP measurements of nanoparticles were performed three times for each sample at 25 °C on a (Malvern Zetasizer Nano ZS (Malvern, UK), with aback scattering detection angle of 173°. Before the analysis, the sonicated dispersion of nanoparticle samples was diluted with ultrapure distilled water, (1 mL of sample in the cuvette), and 0.75 mL in the cuvette (cell) was adequate for ZP.

#### 2.3.3. Transmission Electron Microscopy (TEM) Analysis

The size and morphological study of GNPs, and SGNPs were done in the TEM. 1ml of the sample containing NPs was diluted to 5 mL with ultra-pure water and sonicated in the ultrasonic bath for 10 to 20 min for homogenization; subsequently, a few drops of the NPs suspensions were added to the formvar carbon-coated 200 mesh copper TEM grid and allowed to dry it in the glass desiccator for keeping it secure from contamination. The grid was placed in the specimen holder to be adjusted in the instrument and begin measurement. TEM images of NPs were taken using Transmission electron microscopy—JEOL—JEM–1400 PLUS (Peabody, MA, USA).

#### 2.3.4. Fourier Transforms Infrared Spectroscopy (FT-IR) Study

FT-IR spectroscopy was performed to confirm and analyze the functional groups present on SIL powder as control, GNPs, and SGNPs by the potassium bromide (KBr) pellet method. Prior to analyzing samples by FTIR, (GNPs, and SGNPs) solutions were lyophilized. The KBr was added into an agate mortar and ground to a fine powder [37]. Subsequently, various dried samples were mixed separately with KBr powder at a ratio of 1:10 (sample: KBr). The mixture was ground for 2–4 min and pressed by using a hydraulic press. The mixed powder was then pressed for 1–2 min to form around the pellet. The pellet was cautiously removed and transported to the FTIR sample holder. The measurements were carried out in the range between 400 and 4000 cm^−1^ using Bruker Vector 22 FTIR Spectrometer (Bremen, Germany).

#### 2.3.5. Drug Entrapment Efficiency and Loading Capacity 

SGNPs sample was separated from their aqueous medium containing free drug by centrifugation separation at 10,000 rpm for 15 min. Redispersed in Milli-Q water, then, the concentration of SIL in the supernatant was diluted and analyzed by UV spectrophotometer at = 285 nm (Absorbance was 0.985). A calibration curve of known SIL concentrations vs. absorbances was constructed. The Entrapment efficiency (EE %) and drug loading capacity (LC %) of SIL in SGNPs was calculated by the following formula:

$$\mathrm{EE}\%=\frac{\left(\mathrm{Total}\mathrm{amount}\mathrm{of}\mathrm{SIL}\mathrm{for}\mathrm{preparing}\mathrm{nanoparticles}-\mathrm{free}\mathrm{SIL}\mathrm{in}\mathrm{supernatant}\right)}{\mathrm{Total}\mathrm{amount}\mathrm{of}\mathrm{SIL}\mathrm{for}\mathrm{preparing}\mathrm{nanoparticles}}\times 100$$

$$\mathrm{LC}\%=\frac{\left(\mathrm{Total}\mathrm{amount}\mathrm{of}\mathrm{SIL}\mathrm{for}\mathrm{preparing}\mathrm{nanoparticles}-\mathrm{free}\mathrm{SIL}\mathrm{in}\mathrm{supernatant}\right)}{\mathrm{Nanoparticle}\mathrm{sweight}}\times 100$$

#### 2.3.6. In Vitro Drug Release Study

This method was described by Radu et al. [38]. Briefly, 10 mg of SGNPs dry powder form was dispersed in 10 mL of PBS containing 0.1% tween 20. The solution was incubated in a shaking water bath at 37 °C under shaken horizontally at 250 rpm. At time intervals (0.5, 1, 2, 4, 6, 12, 18 and 24 h), the samples were centrifuged at 10,000 rpm for 15 min, 5 mL of supernatant was withdrawn and replaced by the same volume of fresh dissolution medium at 37 ± 0.5 °C to maintain the overall volume of the sample stable until the end of the experimental period to examine the release kinetics of silymarin drug. The SIL release from SGNPs was evaluated by measuring the absorbance of solutions at 285 nm. Drug release data were adjusted by converting the concentration of drug into a percentage of the cumulative release of drugs according to the following formula [39]:

$$\%\mathrm{of}\mathrm{cumulative}\mathrm{drug}\mathrm{release}=\frac{\mathrm{Released}\mathrm{SIL}\mathrm{from}\mathrm{NPs}\mathrm{at}\mathrm{time}}{\mathrm{Total}\mathrm{amount}\mathrm{of}\mathrm{SIL}\mathrm{in}\mathrm{NPs}}\times 100$$

### 2.4. In Vivo Animals Studies

Thirty male Sprague-Dawley (SD) rats, 3–4 months old, weighing 250 ± 10 gm were used throughout the in vivo experimental work. The animals were purchased from the animal house facility of the Medical Technology Center, MRI, Alexandria, Egypt. Animals have housed in individually ventilated cages under controlled conditions (22 °C, and 12:12 h light/dark cycle) with free access to food and water. Rats were kept under observation for one week preceding the study. All procedures were performed in accordance with the Institutional Animal Care and Use Committee (IACUC)-Alexandria University, Egypt (Approval No.: AU0122132423). The study also follows ARRIVE guidelines and comply with the National Research Council’s guide for the care and use of laboratory animals.

#### 2.4.1. Establishment of Liver Fibrosis Model 

Liver fibrosis was induced in rats with *i.p.* injection with Carbon tetrachloride (CCl_4_) (1 mL/kg) dissolved in olive oil (1:1 *v*/*v*) [40] for 10 weeks; three times weekly, then two times weekly for 2 weeks.

#### 2.4.2. Experimental Design

The animals were divided into five groups (six animals each): control group: received *i.p* injection of olive oil as a vehicle, CCl_4_-group: liver fibrosis was induced by *i.p* injection of CCl_4_ without treatment, SIL-treated group: After the induction of liver fibrosis the rats received a daily dose of SIL (100 mg/kg b.w in olive oil) [41], GNPs-treated: After induction of liver fibrosis the rats received a daily dose of GNPs (20 mg/kg b.w), and SGNPs-treated group: After the induction of liver fibrosis the rats received a daily dose of SGNPs (20 mg/kg b.w) [42]. All treatments were administered by oral gavage for 30 days.

#### 2.4.3. Blood Sample Collection and Tissue Preparation

After the last dose, the animals were kept fasting for 12 h weighted, and anesthetized by deep isoflurane inhalation. The blood samples were obtained by cardiac puncture and centrifuged at 3000 rpm, 4 °C for 20 min to separate the serum. Livers were collected, washed, and weighted to calculate the liver index using the formula:

[Liverweight(g)/Bodyweight(g)]×100

The tissues were divided into three parts; one was fixed in 10% normal formalin for H&E staining, Masson’s trichrome staining (MT), and immunohistochemistry (IHC). The second was frozen at −80 °C for total RNA extraction to assess gene expression. The third part was homogenized in cold 0.1 mM PBS in a ratio (1:9) to be used for ELISA and lipid peroxidation measurements.

#### 2.4.4. Bioinformatic Study

Using literature review over the past 10 years we selected three microRNAs that play important role in the pathogenesis of liver fibrosis. The selected microRNAs are miR-22, miR-29c, and miR-219a. The possible target genes of these microRNAs were identified using the online database; namely, Targetscan (http://www.targetscan.org/) (accessed on 5 October 2020).

### 2.5. Methods

#### 2.5.1. Serum Biomarkers for Liver Function Tests

The activities of alanine aminotransferase (ALT), aspartate aminotransferase (AST), and alkaline phosphatase (ALP), and levels of total bilirubin, and albumin were measured using commercially available kits (Biosystems S.A. Costa Brava 30, Barcelona, Spain) following manufacturer’s instructions. 

#### 2.5.2. Malondialdehyde (MDA) as Index of Lipid Peroxidation

MDA in homogenate was determined according to the method of Draper & Hadley [43]. The sample was heated with thiobarbituric acid (TBA) at low pH (3.5). The resulting pink chromogen has a maximal absorbance at 532 nm.

#### 2.5.3. ELISA Measurements

The hepatic contents of TGF-β1 and Nrf2 in the tissue homogenate were determined using rat specific ELISA kits (Chongqing Biospes Co., Chongqing, China) according to the manufacturer’s instructions.

#### 2.5.4. Gene Expression Analysis

Total RNA was isolated using miRNeasy mini kit (Qiagen, Hilden, Germany) according to the manufacturer’s instructions. The concentration of total RNA was determined using nanodrop (Jenway, Staffordshire, UK). The isolated RNA was reverse transcribed using miScript II RT Kit (Qiagen, Hilden, Germany) according to the manufacturer’s instructions using miScript HiFlex Buffer to promote the conversion of all RNA species into cDNA. 

##### Assessment of Hepatic microRNAs (miR-22, miR-29c, and miR-219a)

The synthesized cDNAs were used for qPCR analysis of mature miRNAs (miR-22, miR-29c, and miR-219a) using Primer Assays (forward primers) and the miScript SYBR Green PCR Kit, which contains the Universal Primer (reverse primer) and QuantiTect SYBR Green PCR Master Mix (Qiagen, Germany). The kit was used with miScript PCR Control U6 (Qiagen, Germany). Quantitative PCR amplification conditions started with an initial denaturation for 10 min at 95 °C and the then amplification by 40 cycles of PCR as follows: Denaturation at 95 °C for 15 s, annealing at 50 °C for 20 s, and extension at 60 °C for 20 sec. Data were collected using Rotor-Gene Q-Pure Detection version 2.1.0 (build 9) (Qiagen, Germantown, MD, USA). 

##### Assessment of Hepatic Expression of TGFβR1, TGFβR2, and COL3A1 Genes

The cDNA was used to quantify the hepatic gene expression of TGFβR1, TGFβR2, and COL3A1 by Rotor-Gene Q qPCR (Qiagen, USA) using QuantiTect SYBR Green PCR Master Mix (Qiagen, Germany). The kit was used with miScript PCR Control GAPDH (Qiagen, Germany). Quantitative PCR amplification conditions started with an initial denaturation for 5 min at 95 °C and the then amplification by 40 cycles of PCR as follows: Denaturation at 94 °C for 15 s, annealing at 55 °C for 20 s and extension at 60 °C for 40 s. Primers used for rat genes were presented in (Table 1).

##### Relative Quantification of Gene Expression

The relative expression of miRNAs and mRNAs were quantified relative to the expression of the reference genes (U6 for miRNAs and GAPDH for mRNAs) in the same sample by normalizing the threshold cycles (Ct) values of target miRNAs and mRNAs to that of U6 and GAPDH, respectively, using the ΔΔCt method. The results were expressed as relative expression ratio or fold-change compared with the ‘Control group’ according to Livak method [44].

### 2.6. Histopathology Study

The samples were dehydrated in gradual ascending ethanol, cleared in xylene, and embedded in paraffin. Five-micron thick paraffin was sliced using a microtome (Leica RM 2155, England) and then routinely stained with hematoxylin/ eosin (H&E) [45]. The fibrous lesion areas were identified using Masson’s trichrome (MT) staining to detect collagen fibers; the collagen deposition of each group was calculated using ImageJ software [46]. All section photos were photographed with (a Leica^®^ microscope, Germany, combined with Am Scope microscope digital camera). Fibrosis staging scores were designed as the following criterion: score (0) absent fibrosis; score (1) slight fibrosis; score (2) mild fibrosis; score (3) moderate fibrosis; score (4) severe fibrosis. The evaluation was described previously [47].

#### Quantitative Measurements of Fibrosis Area

Quantitative analysis of liver fibrosis are (as %) was performed on sections stained with Masson trichrome stain using ImageJ software (Image J 1.47v, National Institute of Health, Bethesda, MD, USA). The color settings in the ImageJ program were preserved with the blue-stained region measurements in the samples at all times, these measurements were performed for images of at least 10 different fields per section at a magnification power of ×100 [46].

### 2.7. Immunohistochemistry (IHC) Study

IHC detection of alpha-smooth muscle actin (α-SMA) as a marker for HSCs activation was performed using avidin-biotin-peroxidase complex (ABC) [48]. Briefly, paraffin-embedded tissue sections of 3µ thickness were deparaffinated in xylene and then rehydrated in a graded series of ethanol and incubated in methanol 0.3% H_2_O_2_ at room temperature for 30 min. The sections were washed in distilled water for 5 min, followed by a rinse in PBS at pH7.3 for 5 min [49], and blocked with 5% of bovine serum albumin (BSA) in PBS and incubated with sections for 1h at room temperature. The sections were incubated with Anti-α-SMA primary antibody at a concentration of 1 g/mL containing 5% BSA in PBS and incubated overnight at 4 °C. α-SMA primary antibody was obtained from (Abcum, Cambridge, MA, USA). The slides were washed 3 times by PBS at pH 7.4, then incubated with Goat Anti-Rabbit IgG H&L (HRP) secondary antibody for 1 h at room temperature, and washed 3 times and incubated for 5–10 min in 0.02% Diaminobenzidine (DAB) containing 0.01% hydrogen peroxide, then counterstained by hematoxylin and the slides were visualized under a microscope. Immunohistochemistry reaction’s positive percentage was counted by using ImageJ software (Image J 1.47v, National Institute of Health, Bethesda, MD, USA).

### 2.8. Statistical Analysis

The data were input into the computer and analyzed with the IBM SPSS software package version 20.0. (IBM Corp., Armonk, NY, USA). The Kolmogorov-Smirnov test was performed to ensure that the distribution was normal. Quantitative data were described using mean and standard deviation. The significance of the obtained results was judged at the 5% level. The F-test (ANOVA) was used for normally distributed quantitative variables and comparing more than two groups, followed by the Post Hoc test Tukey for pairwise comparisons.

## 3. Results

### 3.1. Characterization of Gold Nanoparticles (GNPs)

#### 3.1.1. UV–Visible Spectrophotometry

**Figure 1:**
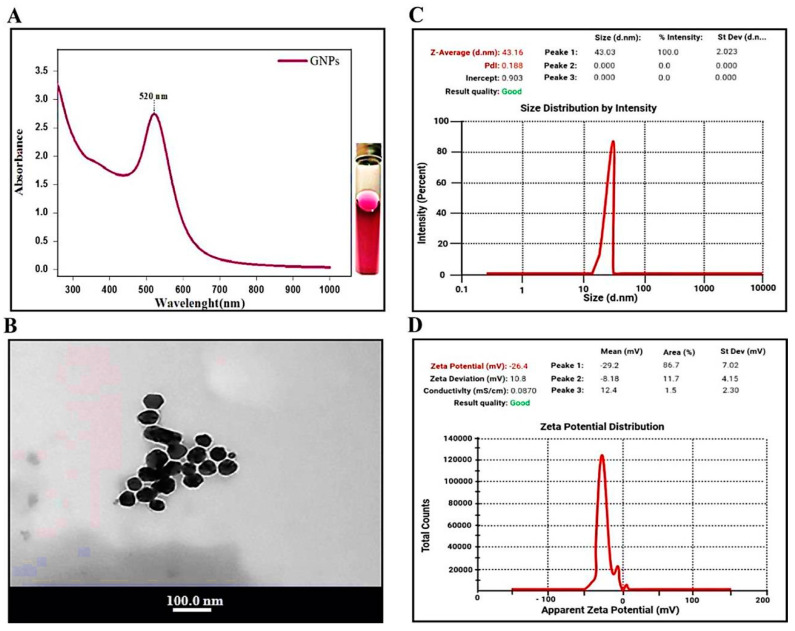
Characterization of Gold nanoparticles (GNPs): (**A**) UV-Visible spectrum. (**B**) Transmission electron microscopy images. (**C**) Particle size distribution of GNPs assessed by dynamic light scattering (DLS), (**D**) Zeta potential distribution of GNPs by dynamic light scattering (DLS).

Optical measurements of functional GNPs verified the identity and functionality of the nanoparticles. GNPs exhibited UV-Vis maximum absorbance values with an optimal peak at 520 nm (Figure 1A).

#### 3.1.2. TEM Analysis of GNPs

The tem images of the synthesized GNPs showed clear crystalline characteristics of the prepared nanoparticles, with various shapes of both spherical- and triangle-shaped mixed nanoparticles. The average particle size of GNPs was found to be 20 ± 4 nm (Figure 1B).

#### 3.1.3. DLS and Zeta Potential of GNPs

Figure 1C represents the particle size distribution of synthesized GNPs by the Sodium citrate reduction method obtained from DLS measurements. The average size of the prepared GNPs was 43.16 nm with a remarkable polydispersity index (PDI) 0.188. 

Figure 1D indicates the corresponding average Zeta potential distribution value of the synthesized GNPs with Sodium citrate (−26.4 mV).

### 3.2. Characterization of Silymarin-Gold Nanoparticles (SGNPs)

#### 3.2.1. UV–Visible Spectrophotometry

**Figure 2:**
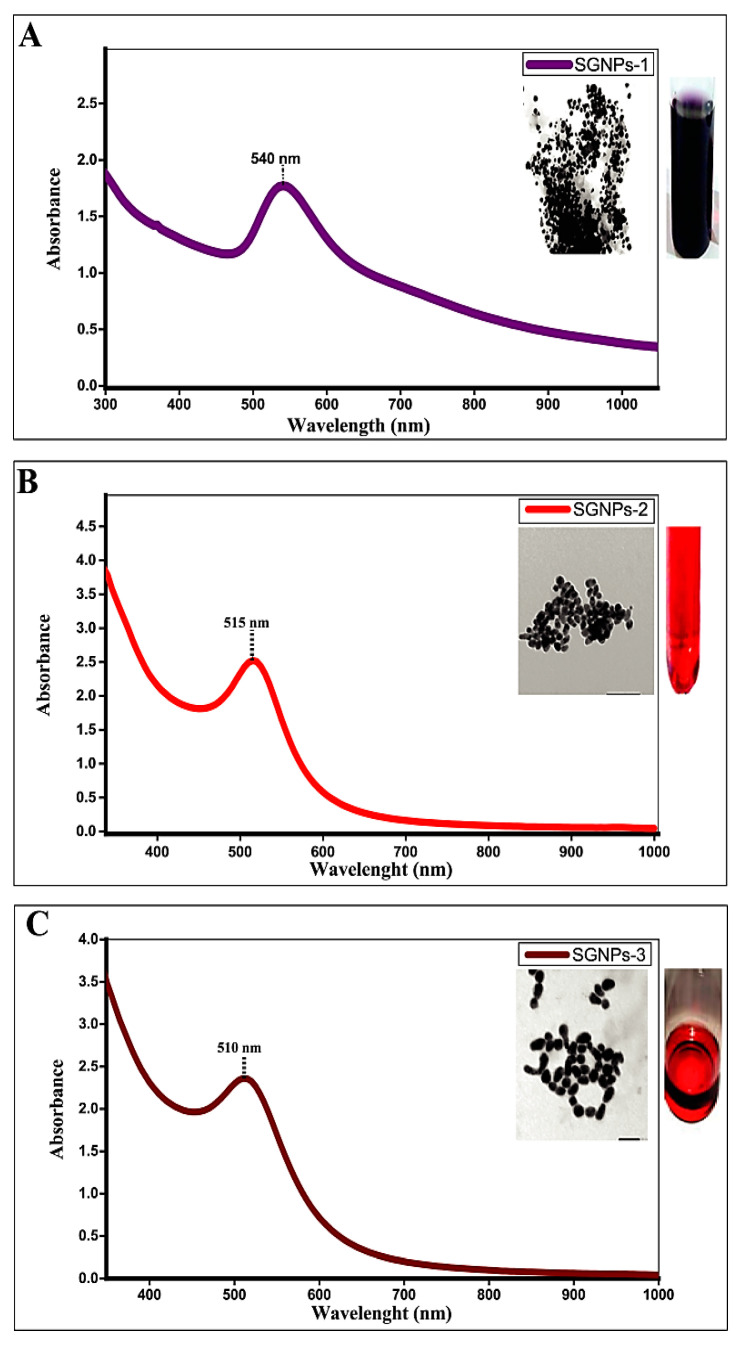
The UV-Visible spectrum of Silymarin-conjugated gold nanoparticles (SGNPs) obtained after the interaction: The absorption peak at (**A**) 540 nm; (**B**) 515 nm; and (**C**) 510 nm, corresponding to the different concentrations of silymarin (1.0. mM/5 mL); (2.0 mM/5 mL); and (2.5 mM/5 mL) respectively.

The successful conjugation of silymarin and gold was visually identified by altering the color of the SIL solution from yellow to different colors (dark violet-red, maroon red, burgundy red). The gradual color change from violet to burgundy red color was observed during the reaction with varying concentrations of silymarin with keeping the constant concentration of HAuCl4, which are characteristics of the SPR of different sizes of SGNPs in solution. The peaks of the SPR spectra were obtained differently depending on the different concentrations of silymarin (1.0 mM, 2.0 mM, 2.5 mM), respectively at a constant concentration of HAuCl4 (1.0 mM); SGNPs exhibited the typical band absorption associated with plasmonic nanoparticles at 540 nm, 515 nm, and 510 nm with average particle size at 19.51 ± 1.74 nm, 18.4725 ± 1 nm, and 16.66 ± 0.5 nm, respectively (Figure 2A,C). The maximal SPR peak of SGNPs showed at 540 nm with the low concentration of SIL (1.0 mM). At higher concentrations (2.0 mM, 2.5 mM), the SPR peaks were shifted towards a shorter wavelength region at 515 nm, 510 nm, respectively, which shows a decrease in particle size. The longer wavelength of SPR at lower concentrations was presumably due to the damping of the SPR induced by the combined effect of increased particle size and shape of the SGNPs in colloidal solutions.

#### 3.2.2. TEM Analysis of SGNPs

**Figure 3:**
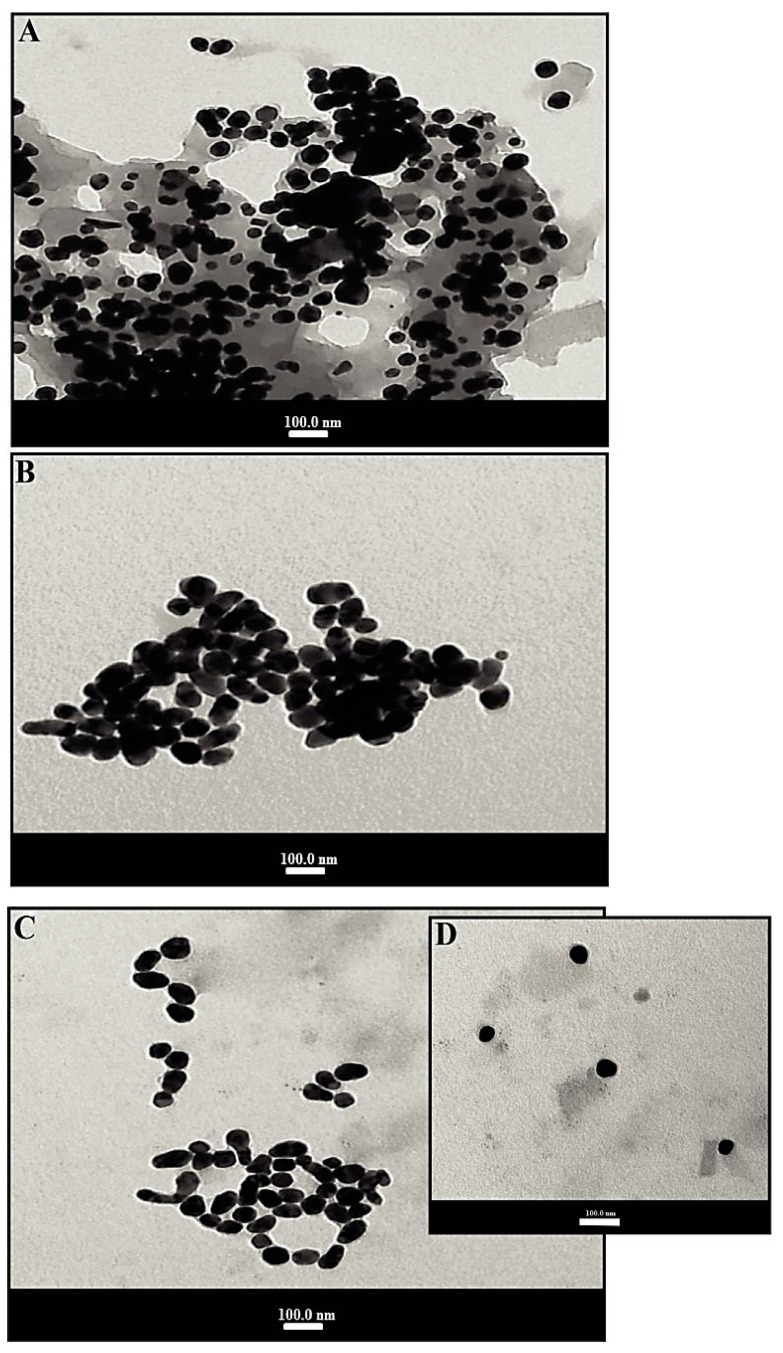
Transmission electron microscopy images of SGNPs: The average particle size (**A**) 19.51 ± 1.74 nm; (**B**) 18.47 ± 1 nm; and (**C**,**D**) 16.66 ± 0.5 nm, corresponding to the different concentrations of silymarin.

TEM was employed to further describe the size, morphology, and structure of resulting SGNPs. Figure 3A,D depicts the TEM images of SGNPs at different scales to confirm the morphology of the colloidal sol. The SGNPs were predominantly spherical but very little anisotropic particles were also seen. TEM results confirmed the crystalline nature of the synthesized nanoparticles. The average particle size of SGNPs was found to be 19.51 ± 1.74 nm, 18.4725 ± 1 nm, and 16.66 ± 0.5 nm, respectively, based on the preparation of SGNPs at different concentrations of SIL. GNPs can be differentiated within these structures where they are densely aggregated, this aggregation being responsible for the redshift on the experimental SPR band.

#### 3.2.3. DLS and Zeta Potential of SGNPs

**Figure 4:**
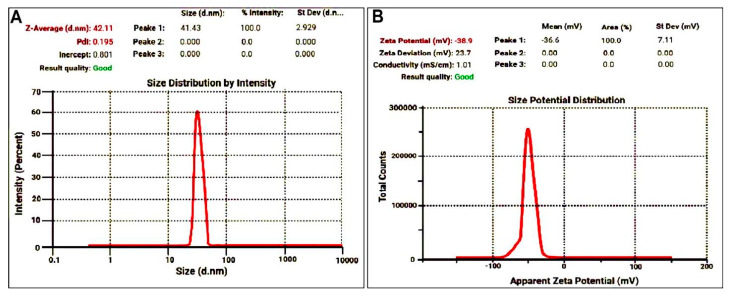
(**A**) The particle size distribution for synthesized SGNPs by dynamic light scattering shows the average size at 42.11 nm; and (**B**) Zeta potential of SGNPs shows stable negative charge at −38.9 mV.

Hydrodynamic diameters of SGNPs were confirmed by DLS. Figure 4A represents the DLS particle size distribution curve with a good PDI of 0.195 with a mean size of 42.11 nm were found. The zeta potential shows the potential stability and surface charge of the colloidal nanocomposite being synthesized. The result of zeta potential value for the synthesized SGNPs is shown in Figure 4B with the presence of a stable negative charge at −38.9 mV.

#### 3.2.4. FT-IR Study

**Figure 5:**
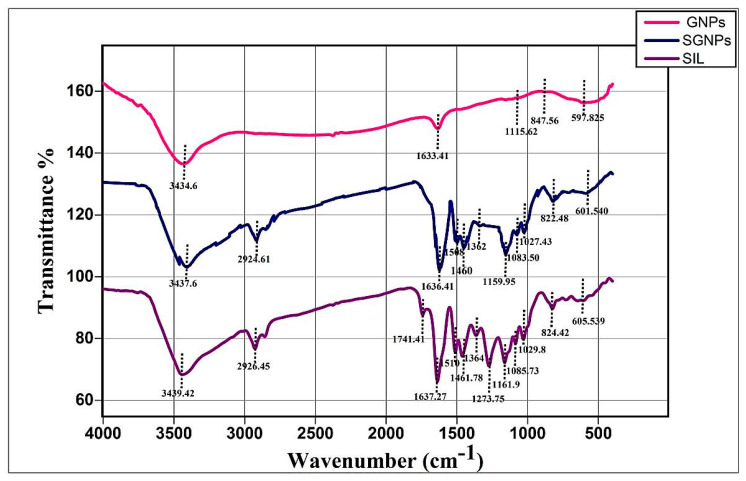
FT-IR spectra of Silymarin (SIL, GNPs, and SGNPs.

Figure 5 shows the FT-IR spectra of pure drug SIL, GNPs, and SGNPs. The spectrum of pure SIL was characterized by group bands at wave numbers 3439.42 cm^−1^ (O–H stretching, phenols/alcohols), 2926.45 cm^−1^ (C–H stretching, alkyl), 1741.41 cm^−1^ (C=O stretching, esters), 1637.27 cm^−1^ (-C=O stretching), 1510–1461.78 cm^−1^ (skeleton vibration of aromatic C=C ring stretching), 1364 cm^−1^ (–C–C–, stretching), 1273.75 cm^−1^ (C–O stretching, polyols), 1161.9 cm^−1^ (C–O stretching, Esters), 1085.30 cm^−1^ (benzopyran ring vibrations), 1029.8 cm^−1^ (C–O group stretching, Sulfoxide), 824.42 cm^−1^ (C-H bending, Alkenes), 605.53 cm^−1^ (C–I stretching, halo compound). These results demonstrated the successful attachment of SIL to GNPs, which is in accordance with UV–Vis, TEM analysis, and DLS measurements.

#### 3.2.5. Drug Entrapment and Loading Capacity of SIL in SGNPs 

The SIL entrapment efficiency from 90% to 96% and loading capacity from 19% to 38.69% was significantly affected by the concentration of silymarin, as shown in (Table 2), and the higher the concentration is, the higher the encapsulation efficiency is.

#### 3.2.6. In Vitro Drug Release Study

**Figure 6:**
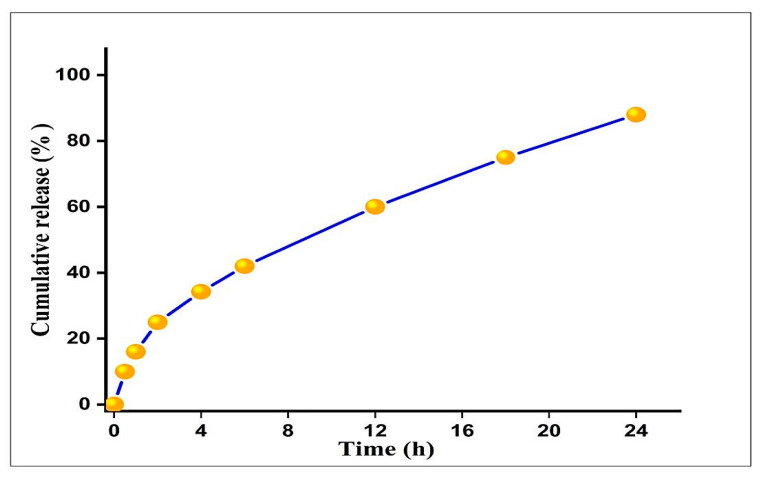
Cumulative in vitro release profile of free SIL from SGNPs. Each data point represented as mean ± S.D (*n*= 3).

As shown in the (Figure 6), the release profile of SIL conjugation GNPs was tested in vitro at 37 °C in PBS at pH 7.4. A typical two-stage release pattern was demonstrated in vitro; the pattern of the first stage release presented a relatively speedy burst and in the early time point (~42% of the entrapped Silymarin release in 6 h) followed by a sustained and incremental phase of release over an extended period of time (~60% to 88% of the drug released for up to 24 h). The detected initial rapid release may be due to the dispersion of a drug present on the surface of SGNPs, accompanied by a slower prolonged release of the SGNPs. 

### 3.3. In Vivo Studies

#### 3.3.1. Body Weight Gain, Liver Weight, and Liver Index (%)

The exposure to CCl_4_ significantly reduced the body weight gain along with an increase in liver weight, and liver index as compared to the control group. CCL_4_-intoxicated rats treated with any of the treatments gained significantly more body weight and had lower values for liver weight and liver index than the CCl_4_-untreated group with the best effect observed in rats treated with SGNPs, which showed completely normal values, while the least effect was observed in CCl_4_-treated rats with GNPs alone (Table 3).

#### 3.3.2. Serum Liver Function Markers

CCl_4_-rats have significantly higher serum activities of ALT, AST, and ALP and bilirubin level and significantly lower albumin level compared with the control rats. The CCl_4_-rats treated with SIL, GNPs, or SGNPs have significant ameliorative effects on all liver serum markers compared with the untreated rats. SIL alone has better effects compared with GNPs. The best effects were observed in the CCl_4_-rats treated with SGNPs which showed no significant changes compared with the control rats (Table 4).

#### 3.3.3. Hepatic Redox Parameters and TGFβ-1

Table 5 shows the hepatic content of malondialdehyde (MDA), Nrf2, and TGF-β. CCl_4_-rats have marked elevation of hepatic MDA and TGF-β levels compared with control rats. The CCl_4_-rats treated with SIL, GNPs, or SGNPs showed significant reduction in the hepatic contents of MDA and TGF-β compared with the untreated rats with the best effect observed in the rats treated with SGNP. SGNPs showed a superior response than SIL and GNPs. On the other hand, the level of nuclear Nrf2 was significantly decreased in the CCl_4_-treated rats compared with control rats. The treatment of CCl_4_-rats with GNPs showed no significant effect on the hepatic content of Nrf2 while SIL treatment mildly but not significantly increases its level. The CCl_4_-rats treated with SGNPs showed significant increase in the Nrf2 level compared with the untreated rats but still lower than the control value.

### 3.4. Molecular Analysis

#### 3.4.1. MicroRNAs Expression

**Figure 7:**
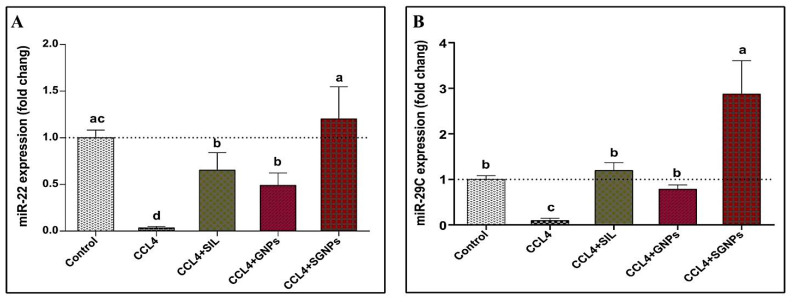

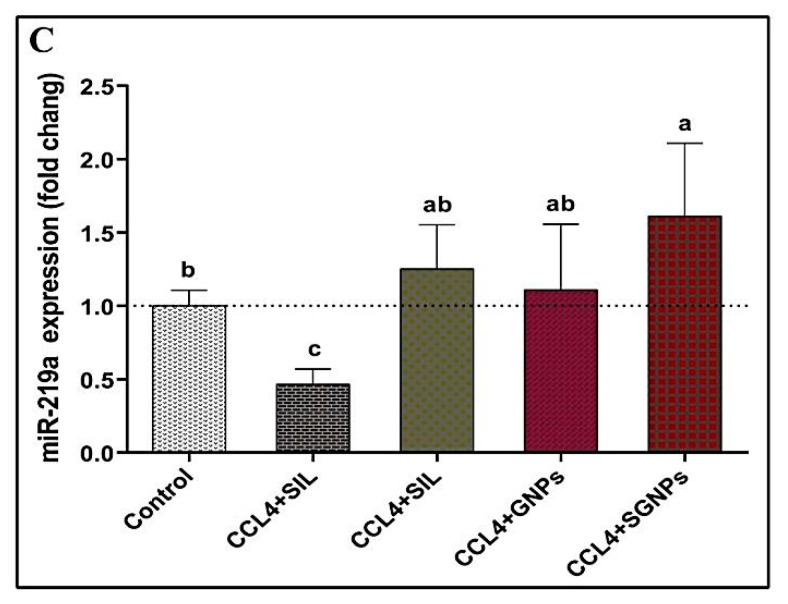
Hepatic expressions of miRNAs: (**A**) miR-22; (**B**) miR-29c; and (**C**) miR-219a. Data expressed as mean ± SD (*n* = 6). Means in the same column with common letters are not significant and means with different letters are significant by ANOVA test followed by Post Hoc Test (Tukey). Statistically significant at *p* ≤ 0.05.

The results of hepatic expression of miRNA-22 are presented in (Figure 7A). CCl_4_- rats showed severe suppression of hepatic miRNA-22 expression, to be about 4% of those in the group of control rats. All treatments +used in the present study significantly preserved the hepatic tissue against CCl_4_-induced suppression of miRNA-22.The best therapeutic effect was observed in the group treated with SGNPs followed by SIL and GNPs.

Hepatic miRNA-29c expression was significantly suppressed in intoxicated rats (about 10% of the control value). The SIL and GNPs treated CCl_4_-rats showed significant up regulation in the expression of miR-29c compared with the untreated rats with no significant difference compared with the control rats. The CCl_4_-rats treated with SGNPs showed significantly higher expression levels compared with the control values (Figure 7B).

The miRNA-219a showed significant downregulation in the CCl_4_-rats compared with the control group. The treatment of CCl_4_-rats with any of the used regimen significantly normalize the expression of miR-219a with the most pronounced effect observed in the rats treated with SGNPs which showed significant higher expression level compared with the control rats (Figure 7C).

#### 3.4.2. Bioinformatics

**Figure 8:**
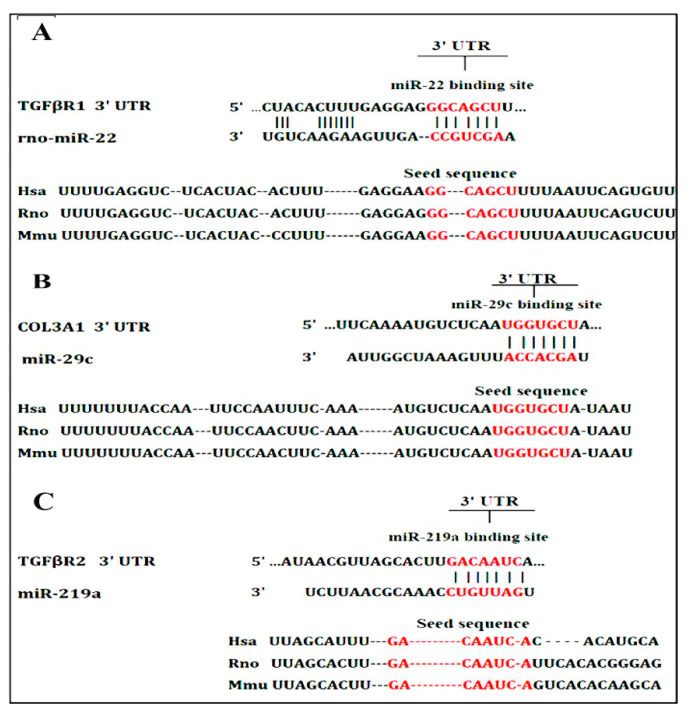
Schematic descriptions of the hypothesized duplexes formed by the interaction between the binding sites in 3′-UTR: (**A**) TGFβR1 and miR-22; (**B**) COL3A1 and miR-29c; and (**C**) TGFβR2 and miR-219a. The seed recognition sites are identified and all nucleotides in these regions are highly conserved across several species, including humans, rats, and mice.

Computational prediction of miRNA targets is an important step for exploring the miRNA-mRNA interactions. After obtaining the sequence microRNAs from the miRBase online tool (http://www.mirbase.org/) (accessed on 10 March 2021). Targetscan online tool (http://www.targetscan.org/) (accessed on 10 March 2021) was used to predict the targets of miR-22, miR-29c and, miR-219a, then we perform functional enrichment analysis of these targets using different bioinformatics tools. In this study, Figure 8A,C shows the predicted interactions between miR-22, miR-29c, miR-219a, and the target sites within the 3′-UTR region of TGFβR1, COL3A1, TGFβR2, respectively.

#### 3.4.3. The Expression of the Target Genes

**Figure 9:**
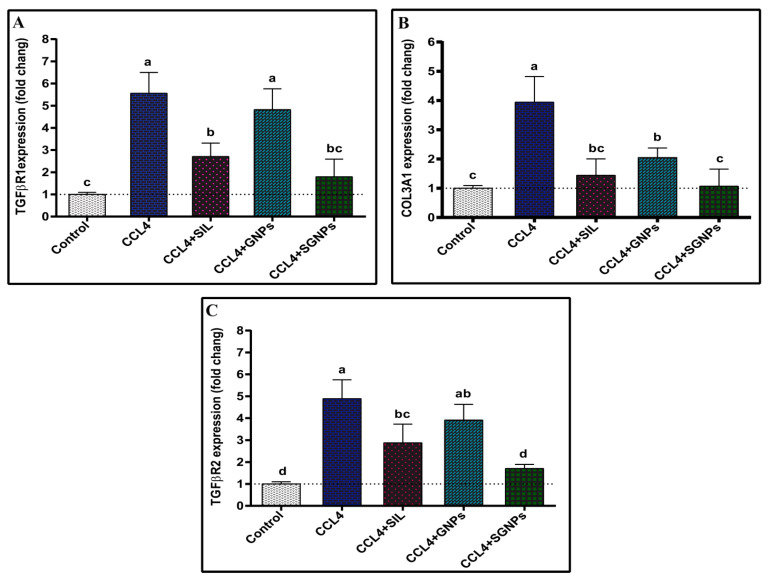
Hepatic expression of target genes: (**A**) TGFβR1; (**B**) COL3A1; and (**C**) TGFβR2. Data expressed as mean ± SD (*n* = 6). Means in the same column with common letters are not significant and means with different letters are significant) by ANOVA test followed by Post Hoc Test (Tukey). Statistically significant at *p* ≤ 0.05. Abbreviations: TGFβR1, transforming growth factor-beta receptor I; COL3A1, collagen type III alpha 1; TGFβR2, transforming growth factor-beta receptor II.

The expression of TGFβR1 was markedly upregulated in CCl_4_-rats compared with control rats. The treatment of CCl_4_-rats with GNPs showed no significant effect while SIL treatment significantly downregulated the hepatic expression of TGFβR1 in CCl_4_-rats compared with the untreated rats. The best ameliorative effect observed was in SGNPs treated rats which normalized the expression (Figure 9A).

The CCl_4_-rats showed significant induction of COL3A1 hepatic expression compared with the control rats. The treatment of CCl_4_-rats with SIL or GNPs significantly suppresses the expression of COL3A1 compared with untreated rats whereas treatment with SGNPs therapy significantly and completely normalizes its expression (Figure 9B).

CCl_4_-rats showed a significantly enhanced hepatic expression pattern of TGFβR2 compared with the control group. The treatment of CCl_4_-rats with GNPs showed no significant effect while SIL treatment significantly downregulated the expression of TGFβR2 compared with the untreated rats. The CCl_4_-rats treated with SGNPs showed nearly normal expression levels (Figure 9C).

#### 3.4.4. Correlation Studies

**Figure 10:**
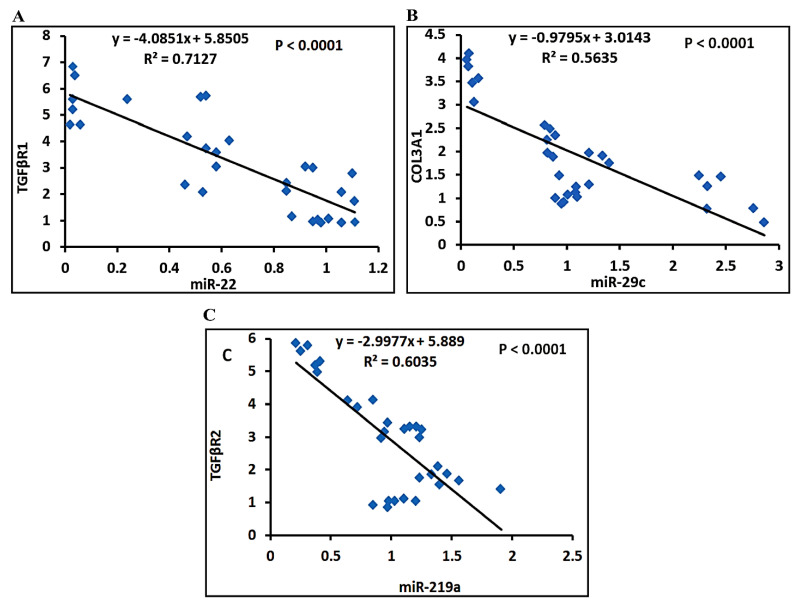
The Pearson significant correlations between miRNAs genes and their target genes: (**A**) correlation curve between miR-22 and TGFβR1 expression; (**B**) correlation curve between miR-29c and COL3A1expression; and (**C**) correlation curve between miR-219a and COL3A1 expression in the hepatic of rats treated with different study groups.

The statistical analysis using Pearson correlation reveals that expression levels of miRNAs were inversely correlated with their targets. MiR-22 is inversely correlated with TGFβR1 (Figure 10A), miR-29c is inversely correlated with COL3A1 (Figure 10B), and miR-219a is inversely correlated with TGFβR2 (Figure 10C).

### 3.5. Histopathological Analysis

#### 3.5.1. Liver Morphology

**Figure 11:**
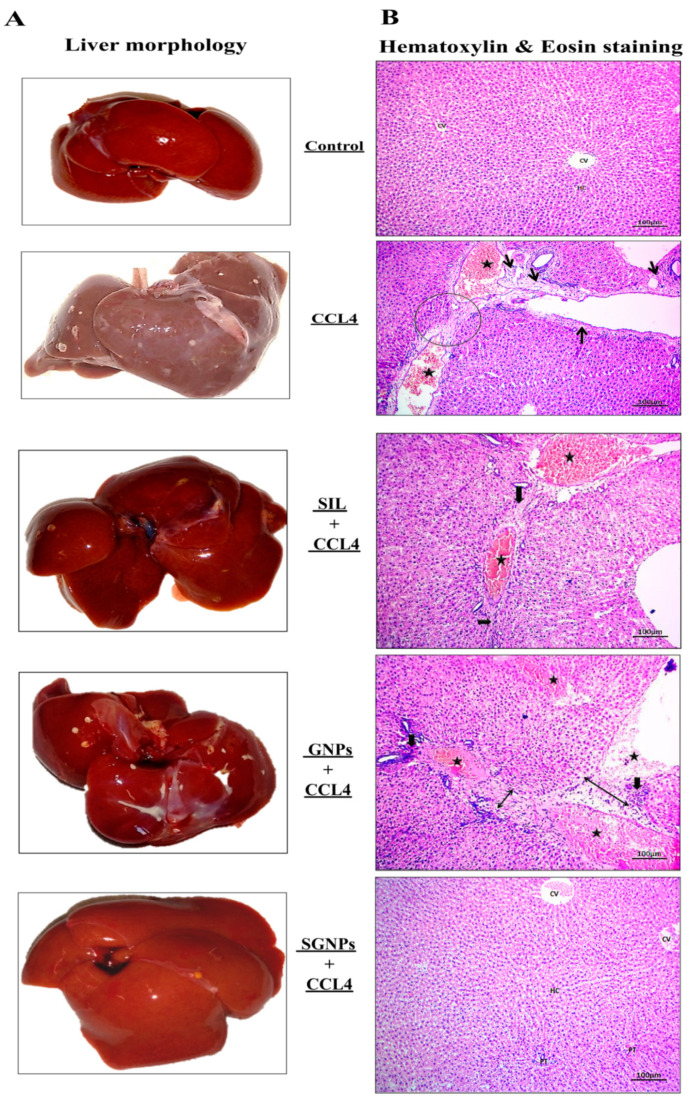

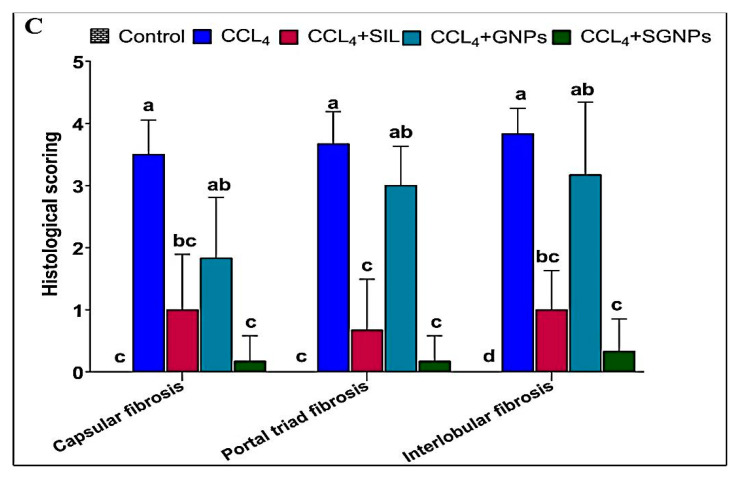
(**A**) Representative photographs of the liver after excision from the animals after the experimental period; (**B**) hematoxylin and eosin-stained liver sections under the microscope; and (**C**) histologic scoring of H&E-stained sections for (interlobular fibrosis, portal triad fibrosis, and capsular fibrosis) as essential and associated lesions indicators for liver fibrosis among treatment studied groups. Data were expressed by using mean ± SD. Means with common small letters are not significant and means with different letters are significant.

Figure 11A shows that the control group treated with olive oil displayed a healthy liver with a soft, smooth, and glossy surface texture. In CCl_4_-rats, the animal’s liver showed a nodular appearance with a rough, stiff, hard patchy surface, and no shine, reminiscent of liver fibrosis in humans. Mild abnormalities in liver morphology were observed in the rats treated with GNPs. Rats treated with SIL were relatively healthy; the livers in this group showed less nodular changes and a smoother surface than those in the CCL_4_-untreated group. The livers of rats in the group that received SGNPs had much improved gross appearance and were comparatively healthier, with bright, shiny, and smooth surfaces.

#### 3.5.2. Histopathology of Liver Tissue

The livers sections of control rats revealed normal appearances in all hepatic architectures, including central vein and hepatic cords, hepatocytes, sinusoids, and portal triad. The liver sections from CCl_4_-rats showed marked widening in hepatic vein and congested blood vessels associated with marked portal fibrosis which contains mature collagen fibers deposited and fibroblasts hyperplasia. The treated groups demonstrated variable degrees of the ameliorative effects of the previous alteration. The SIL treated rats showed slight improvements with the presence of slight portal fibrous strands, additionally, blood vessels were congested accompanied by regenerated hepatic structures. The liver sections of GNPs treated rats showed interlobular fibrosis, massive degenerated hepatic lobules with multifocal inflammatory cell aggregations, and marked congested blood vessels. Finally, liver sections of the rats treated with SGNPs revealed a return to normal hepatic architectures with still slight congested blood vessels in the portal trade and mild kupffer cell hyperplasia (Figure 11B).

#### 3.5.3. Histological Grading of Fibrosis

The degree of histological changes in the liver (interlobular fibrosis, portal triad fibrosis, and capsular fibrosis) from 0 to 4+ grades among studied groups was described in (Figure 11C) as indicators for liver fibrosis.

#### 3.5.4. Masson’s Trichrome (MT) Staining of Liver Tissue

**Figure 12:**
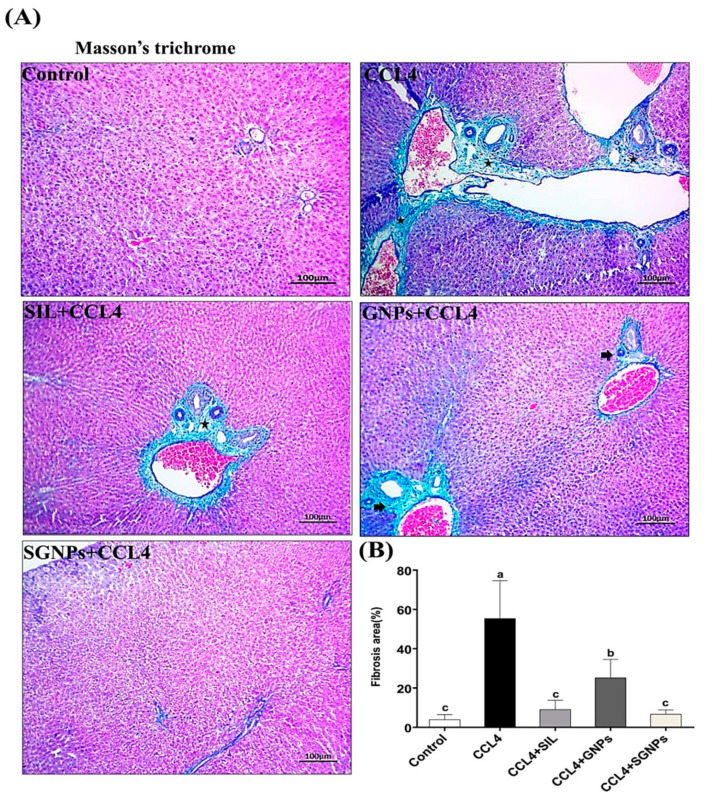
(**A**) Mason’s trichrome stained liver sections under microscope; CCl_4_-rats show marked intense blue satiable materials surrounded the bile ducts and blood vessels in the portal area indicating prominent fibrosis, however other groups show signs of fibrosis unevenly except SGNPs, which show a significantly decreased degree of liver fibrosis. (**B**) Fibrosis score of different groups as calculated by Masson’s trichrome staining (MT) area percent (fibrosis area % per 10 fields in magnification power ×100) using ImageJ software. Data are expressed as mean ± SD (*n* = 6). Means in the same column with common letters are not significant and means with different letters are significant by ANOVA test followed by Post Hoc Test (Tukey). Statistically significant at *p* ≤ 0.05.

No collagen deposition was shown in the normal liver when stained with MT. In CCl_4_-rats, MT staining revealed the accumulation of matured collagen fibers (stained blue) (Fibrosis model). The liver sections from CCl_4_-rats treated with SIL showed a decrease in these changes with fewer fibers compared with the untreated rats. The rats treated with GNPs had more fibers than the SIL group. On the other hand, the best findings were observed in animals treated with SGNPs which showed apparently normal hepatic architecture, and a significant decrease in the degree of liver fibrosis when compared with SIL and GNPs as monotherapy (Figure 12A,B).

#### 3.5.5. Immunohistochemistry (IHC) of αSMA

**Figure 13:**
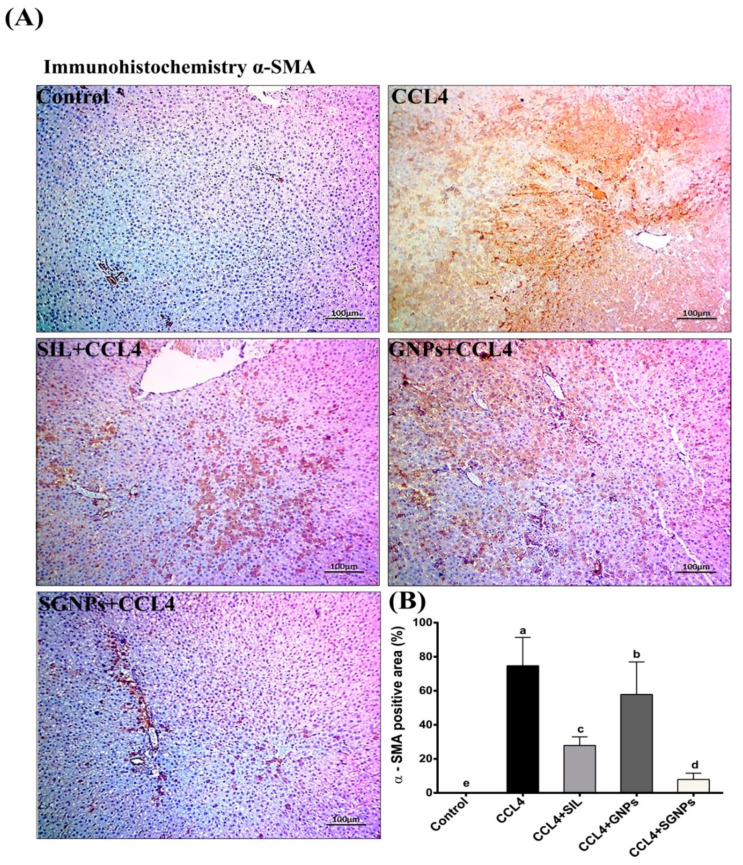
(**A**) Immunohistochemistry staining for α-SMA in liver sections; In the case of CCl_4_-rats, the liver section exhibited high immunoreactivity; however, the number of α-SMA immunoreactive cells was significantly decreased by treatment groups of SIL, GNPs, and SGNPs, with the best result in SGNPs group, which showed a potent response in the attenuation of immunohistochemistry reactions. (**B**) α-SMA immunohistochemistry reactions positive area (average number of positive cells per 10 field’s high power ×400) quantified with Image J. Data are expressed as mean ± SD (*n* = 6). Means in the same column with common letters are not significant and means with different letters are significant by ANOVA test followed by Post Hoc Test (Tukey). Statistically significant at *p* ≤ 0.05.

In the control group, there were no positive α-SMA cells and no immunohistochemistry reactions. The livers in CCl4-rats showed a high expression of α-SMA immunoreactivity. α immunoreactive cells were reduced dramatically by treatments with the exception of the GNP-treated group, where α-SMA-positive cells in the liver were significantly increased in comparison with the control group. SIL-treated rats showed moderate α-SMA immunoreactive cells compared with the untreated rats. Interestingly, the livers of rats who received SGNPs treatment showed a staining pattern similar to control animals with sporadic α-SMA positivity (Figure 13A,B).

## 4. Discussion

This work was aimed to prepare and characterize silymarin-loaded gold nanoparticles formulations (SGNPs) to improve the antifibrotic effects of SIL in a rat model of CCl_4_-induced liver fibrosis. The results confirmed the successful synthesis of SGNPs and were characterized using UV–visible spectrophotometry, TEM, DLS, and FT-IR. Most of the formulated SGNPs were spherical with few inhomogeneity particles and distributed with sizes ranging from 16 up to 20 nm. The explanation for the forming of anisotropic NPs can be based on the following; the stability of spherical nanoparticles formed at the start of the reaction was due to the adequate availability of protecting biomolecules. On the other hand, owing to the lower abundance of protective molecules, particles produced later were less stable; these budding nanocrystals lack protective molecules, thus thermodynamically unstable [50]. The inspection of TEM micrographs revealed that the synthesized SGNPs were not in close interaction within the aggregates, suggesting the stability of the NPs [51]. The coexistence of SGNPs in small and large sizes was attributed to the SGNPs formed in the initial and later periods of the reaction, which indicates that both nucleation to form new NPs and the aggregation to form larger particles occurred sequentially [51]. Although GNPs are considerably less toxic than other metal NP, GNPs have been documented to have size-related toxicity as the particle size < 2 nm demonstrated higher toxicity relative to larger particle size [52]. The spherical GNPs of size 10–20 nm displayed less cytotoxicity and more bacteriostatic properties [53]. In this study, the mean hydrodynamic diameters of SGNPs measured by DLS are 42.11 nm. The Z-average diameter of the metallic NPs is typically greater than the core size of the particle, as the DLS measurement is done to measure the thickness of the protective shell of the cap or stabilizing agent enveloping the metallic NPs, the hydration layer, and the actual size of the metallic core [54]. The relatively large size of SGNPs observed using DLS demonstrated that the drug is attached to the surface of gold nanoparticles. The ratio of particles of various sizes to the total number of particles is described by polydispersity (PDI). The higher the PDI value, the less the nanoparticles are monodispersed [55]. In this study, The PDI value reflects, 0.195, monodispersity, and perfectly dispersed of the SGNPs without aggregation [56]. 

The zeta potential reading in the current study was a high electronegative value (−38.9 mV), which corresponds to gold nanoparticles that were negatively charged and indicated that the loaded nanoparticles were stable as illustrated in the guideline [57]. Zeta potential measurement is an important physicochemical predictor of the stability of nanoparticles. Higher positive or negative values result in greater repulsive strengths, while repulsive forces between particles with similar charges help prevent the aggregation of particles, and thus facilitate re-dispersion. Not only does the negative surface charge of gold nanoparticle suspensions suggest high stability, but it also suggests less toxicity to normal cells [58]. It is widely recognized that the positively charged NPs are more internalized by cells than those which are negative or neutral. This higher intracellular build-up contributes to speed deterioration of cell integrity and thus leads to increased cell toxicity. Conversely, anionic or neutral NPs have a lower affinity for cells compared to positively charged ones [59]. This could predict the low toxicity of the silymarin- synthesized GNPs that are negatively charged particles. The development of stable aqueous dispersions and uniform size of gold nano-colloids using natural non-toxic reducing agents is a crucial point for future medicinal applications without costly purification procedures or unwanted side reactions.

FT-IR measurements showed the existence of a similar silymarin spectrum in SGNPs with minor shifts in distinctive wavenumbers indicated the conjugation of the flavonoid to the gold NPs. furthermore, the 1273.73 cm^−1^ band, in specific, most likely arises from the C–O polyol group. The full absence of this band after bioreduction may be attributed to the fact that polyols are predominantly responsible for the reduction of Au III ions [60]. The evidence confirmed the formation of SGNPs and this was consistent with previously reported findings for SGNP [61].

The entrapment efficiency and loading capacity of SIL are increased from 90% to 96% and 19% to 38.69%, respectively, by increasing the concentration of silymarin from 0.482 mg/mL to 1.2 mg/mL. This may attribute to the presence of a sufficient amount of HAucl4.H2O to trap silymarin. SGNPs exhibited a biphasic release profile in a releasing medium with a pH value of 7.4 were determined using UV-visible spectroscopy assay. After 6 h, ~42% of the SIL was released followed by a sustained ~60% to 88% of the drug release up to 24 h. SIL’s biphasic release was probably attributed to nanoparticle surface-adsorbed molecules and SIL’s poor water solubility [62]. Thus, the results showed that SGNPs were able to particularly stimulate the delivery of the silymarin to liver fibrosis [63].

The anti-fibrotic effects of SGNPs in comparison with SIL solution and GNPs were assayed in vivo on male rats with CCl_4_- induced hepatotoxicity. The model of rat liver intoxication with CCl_4_ is a suitable approach used for the screening of the therapeutic effects of many herbs and novel drugs [64].

CCl_4_ is a well-known hepatotoxin that is used widely to study the induction of toxic hepatic injury in laboratory animals. The CCl_4_-induced damage is the analogue of hepatic damage caused by the hepatotoxins in humans and CCl_4_-induced cirrhosis shares several characteristics with human cirrhosis of different etiologies; so, it is a suitable model of human cirrhosis [65]. The injury is dependent upon cleavage of the carbon–chlorine bond and peroxidative decomposition of cytoplasmic membrane lipids [66].

The results of the present study indicated increasing hepatic fibrosis with the administration of CCl_4_. At the histopathological level, the liver tissues showed widening in the hepatic vein and congested blood vessels associated with marked portal fibrosis and fibroblasts hyperplasia with an accumulation of matured collagen fibers and high immunoreactivity against α-SMA. These changes are associated with marked weight loss, hepatomegaly, and elevations in the serum bilirubin level, and activities of ALT, AST, and ALP, and a significant drop in albumin level. These results were in accord with numerous studies that indicated alteration of biochemical parameters as biomarkers of hepatotoxicity by CCl_4_ [67,68].

Oxidative stress is the initiating factor of CCl_4_-induced liver fibrosis by inducing hepatocyte injury, inflammation, synthesis of collagen, and activation of HSCs in the liver [69,70]. MDA is a widely used biomarker for the evaluation of lipid peroxidation. Consistent with the previous studies, MDA level was markedly elevated in liver tissue upon exposure to CCl_4_. This may result from the peroxidation of hepatocyte cell membrane phospholipids by reactive oxygen species (ROS) induced by metabolites of CCl_4_ during its biotransformation in the liver [70]. The resultant oxidative stress causes damage to mitochondria, suppresses the mitochondrial electron transport chain [71]. Finally, hepatocyte degeneration and necrosis occur, resulting in the deposition of the extracellular matrix leading to the progression of fibrosis or cirrhosis.

The hepatic content of Nrf2 was pronounced reduced in CCl_4_-rats, which is the master regulator of cellular redox homeostasis that can serve as a sensor of oxidative stress in CCl_4_- induced liver fibrosis [72]. Several studies have shown that stimulation of Nrf2 greatly inhibited liver fibrosis, suggesting that Nrf2 is a potential target for the treatment of liver fibrosis [73,74].

The CCl_4_-rats have a marked increase in hepatic TGF-β1. Several studies have reported induction of TGF-β1 production in serum and liver tissues of CCl_4_-rats models [75,76]. TGF-β1 is a critical player in the pathogenesis of chronic liver diseases [77], and one of the primary profibrogenic mediators [78], that is generally bound to its receptor; TGFβR1, followed by TGFβR2 [79], then the activated TGFβR1 phosphorylates Smad2/3 that initiates the TGF-β/Smads signaling pathway [80]. The elevated TGF-β1 levels as a result of the chronically damaged hepatocytes results in massive cell death.

The documented histological and biochemical alterations in rats with CCl_4_ induced fibrosis were associated with marked molecular changes including marked suppression of miR-22, miR-29c, and miR-219a and induction of their predicted target genes; TGFβR1, COL3A1, and TGFβR2, respectively.

In line with this data, miR-22 was down-regulated in liver samples from patients with liver fibrosis or cirrhosis [81]. Also, Gjorgjieva and her co-workers have found that deficiency of miR-22 dramatically aggravated fat mass accumulation, hepatomegaly, and liver steatosis in mice [82]. The computational analysis suggested TGFβR1 as a potential target of miR-22 which was confirmed in the present study by the marked up-regulation of TGFβR1 in CCl_4_-rats (about 5.5- Fold) and the strong negative correlation between the hepatic expression of miR-22 and TGFβR1 mRNA. TGFβR1 expression was increased in fibrotic livers of humans and rats and its level was correlated with the proliferation of HSCs and increased fibrosis [83]. The study of Wang and his co-workers found that miR-22 could target TGFβR1 and then regulate the downstream SMAD3 signaling pathway [18] which inhibits fibrosis. In line with this, miR-22 can inhibit the expression of TGFβR1 at the posttranscriptional level [84], and able to suppress proliferation and promoting the differentiation of C2C12 cells by targeting TGFβR1 in the myoblast proliferation model [18]. These data underscored miR-22 as a protective and/or therapeutic target of liver fibrosis.

The downregulation of miR-29c was associated with chronic liver inflammation and fibrogenesis in animal models and humans [16]. On the other hand, the hepatic expression of COL3A1 gene (the predicted target of miR-29c) was significantly upregulated in CCl_4_-rats (about 4-fold) and its expression is inversely correlated with miR-29c expression. Previous study demonstrated that miR-29c directly targets COl3A1 mRNA expression in goose fatty liver, where target gene expression was increased in goose fatty liver with a strong inverse correlation with miR-29c [85]. COL3A1 encodes collagen α-1(III) chain, a precursor of collagen III [86], which serves as a ‘cell-binding’ of tissues, the induction of COL3A1 expression plays a crucial role in the drastic enlargement of liver fibrosis in rats. The increased expression of COL3A1 after CCl_4_ administration of rats was reported [87].

The third and least affected microRNA in the present study is miR-219a which showed about 50% suppression in the liver of CCl_4_-rats. Leti and his colleagues observed down-regulated hepatic expression of miR-219a in patients with non-alcoholic fatty liver disease (NAFLD)-related fibrosis [88]. Additionally, miR-219a-5p was suppressed in alcoholic liver disease (ALD) [89]. The present study identified TGFβR2 as a predicted target gene of miR-219a which showed marked upregulation in CCl_4_-rats compared with control rats and its expression was inversely correlated with miR-219a. Mohseni et al., reported upregulated expression of TGFβR2 in the liver tissue of rats received CCl_4_ [90]. TGF-β ligand binding is known to enhance the production of TGFβR2 dimers which stimulate phosphorylation and activation TGFβR1 [17] that ultimately change gene expression, leading to a fibrotic receptive response [91].

Despite significant progress in understanding the pathogenesis of hepatic fibrosis, no antifibrotic treatment is currently licensed for human use. Several potential agents and antifibrotic molecules such as silymarin, caffeine, and curcumin have shown antifibrotic properties [92,93]. In the present study, we formulated and synthesized SIL- loaded Gold nanoparticles (SGNPs) in order to enhance the anti-fibrotic efficiency of SIL. The present study clearly indicated that SGNP significantly boost the anti-fibrotic of SIL in CCl_4_-rats

SIL treatment significantly improved the rate of body weight gain, liver weights, and liver index while SGNPs completely normalized these parameters. On the other hand, GNPs alone have no significant effects. The restoration of previous parameters may reflect the therapeutic effects of SIL and SGNPs against CCl_4_ intoxication and may rely on the elimination of CCl_4_ and its metabolites from the liver [94].

At the histological level, the treatment of CCl_4_-rats with SIL or SGNPs improved histopathological parameters and fibrosis scores and SGNPs showed a pronounced therapeutic effect against chronic liver fibrosis with normal liver architecture comparable to the control group with negligible inflammatory infiltrate. SGNPs have more potent anti-fibrotic potential than SIL alone. Also, Masson’s trichrome staining showed that SGNPs treatment resulted in lesser collagen deposition in the liver than SIL treatment. Furthermore, the immunohistochemistry staining of α-SMA demonstrated the antifibrotic effects of SIL and SGNPs treatments as they showed a staining pattern similar to control animals with a more potent response for SGNPs than SIL and NPs alone. These effects are associated with significant amelioration in the serum parameters including AST, ALT, ALP, bilirubin and albumin. Most of these parameters were completely normalized with SGNPs treatment. Our study was supported by previous studies [95,96].

SIL is known to neutralize the free radicals, prevent oxidative damage, attenuate inflammatory mediators and stabilize the hepatocytes’ cell membrane [97]. SGNPs showed significantly better therapeutic effects than conventional drug SIL and the nanocarrier GNPs which also showed a significant ameliorative effect on liver fibrosis. Gold compounds have the ability to inhibit liver fibrosis and the hepatitis C virus (HCV) [98]. Studies demonstrated that GNPs play an antioxidant and hepatoprotective role against murine hepatic schistosomiasis [99], and have anti-inflammatory, anti-oxidative stress, and anti-fibrosis effects in rats with liver injury [100].

The treatment with SIL or SGNPs produced potential antioxidant effects manifested by a significant decline in the MDA hepatic content and elevation in the NRF2 contents compared with the CCl_4_-rats with the best effects in SGNPs-treated rats. Also, GNPs showed a mild but significant decline in the hepatic MDA and lack a significant effect on NRF2 level. The antioxidant properties of SIL and GNPs have been reported in previous studies; Refs. [101,102] respectively. The anti-fibrotic effect of SIL or SGNPs against liver fibrosis may be mediated through activation of the NRF2 pathway to inhibit oxidative stress-mediated hepatocyte damage.

At the inflammatory level, the treatment of CCl_4_-rats with SIL, GNPs, and SGNPs significantly declined the high hepatic TGF-β1 level with the best effect in the rats treated with SIL and SGNP. This could indicate the ability of these treatments to inhibit HSC stimulation by reducing TGF-β1 production. Previous studies have indicated that the incorporation of SIL with GNPs ameliorates hepatic damage through downregulating liver stellate cells and attenuation of Kupffer cells [35]. Also, the injection of GNPs into schistosomiasis-infected mice resulted in a significant downregulation of IL-1β and TNF-α expressions in the hepatic tissue of mice, thus improving liver dysfunction [99].

At the molecular level, the treatment of CCl_4_-rats with SIL or SGNP resulted in pronounced changes in the hepatic expression of the three studied miRNAs (miR-22, miR-29c, and miR-219a) and their target genes (TGFβR1, COL3A1, and TGFβR2), respectively. The suppressed hepatic expression of miR-22, miR-29c, and miR-219a in CCl_4_-rats were significantly normalized with SIL or SGNPs treatments and even become significantly higher than the control values in the rats treated with SGNPs. On the other hand, the enhanced expression of the target genes TGFβR1, COL3A1, and TGFβR2, respectively, in CCl_4_-rats were significantly downregulated with SIL treatment and completely normalized in the rats treated with SGNPs.

The enhancement of liver miRNAs expression may be a key event in the therapeutic effects of SIL and SGNPs against CCl_4_-induced liver damage. SGNPs demonstrated superior effects in the microRNAs and their targets than SIL. Several studies have demonstrated that SIL has hepatoprotective effects on rat liver damage by increasing the levels of miR-122, miR-192, and miR-194 levels, which contributed to ameliorating liver damage [103]. Furthermore, in the liver of obese diabetic rats, SIL reduced the expression of the pro-fibrotic genes [104]. Also, SIL ameliorates myocardial fibrosis in rats by inhibiting TGF-β1/SMAD signaling involved in TGFβR1 and TGFβR2 expression activation [105].

The mechanism(s) of the epigenetic effects of SIL, or SGNPs is/are unclear; however, many pieces of evidence confirm their epigenetic effects. Silibinin, the major active constituent of the silymarin, induced epigenetic alterations in human prostate cancer cells that involve an increase in the activity of total DNA methyltransferase (DNMT) while decreasing expression levels of histone deacetylases 1-2 (HDACs1-2) [106]. Also, gold nanoparticles targeted the epigenetic pathway for acute myeloid leukemia therapy, by targeting the NCL/miR-221/NFkB/DNMT1 signaling pathway [107].

**Figure 14:**
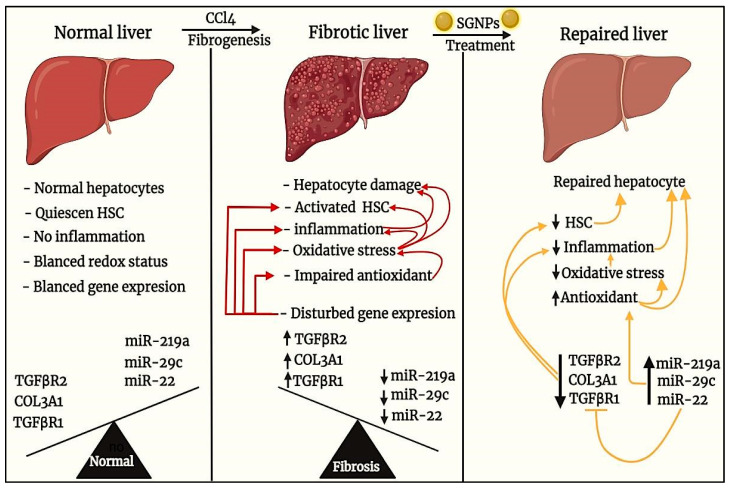
This diagram illustrates and summarizes the anti-fibrotic effects of SGNPs in treating liver fibrosis mediated by CCl_4_.

From the above discussion, we can schematize the anti-fibrotic effects of SGNPs as indicated in (Figure 14). We assume that the main effect is mediated at the molecular level through the induction of hepatic miRNAs (miR-22, miR-29c, and miR-219a) which may inhibit the expression of their targets including the main genes involved in the pathogenesis of liver fibrosis (including TGFβR1, COL3A1, and TGFβR2) and the inflammatory mediators resulting in boosting antioxidant potential, inhibition of fibro-genesis and improving hepatocyte structure and functions. However, the direct effect of SGNPs on the expression of miRNAs needs to be confirmed by the reporter assays which are the main limitation of our study. Also, further studies are needed to investigate the pharmacodynamics and pharmacokinetics of SGNPs and to confirm their molecular mechanism of action.

It’s clear that the SGNPs formulation has more potent anti-fibrotic efficiency than the SIL solution. This discrimination can be explained by the fact that nano-drug formulations are more useful than the usual low-molecular-weight drugs in many ways. The used nano-carrier protects the SIL, enhancing solubility and bioavailability, reducing renal elimination and hepatic degradation, leading to extended circulation time. Furthermore, the gold nano-formulation may increase SIL concentrations at the pathological target, thereby improving the efficacy and toxicity [108], in addition to the biological effects of the nano-carrier itself. The GNPs alone have many hepatoprotective and therapeutic effects as documented earlier [100].

## 5. Conclusions

We successfully synthesized SIL-loaded GNPs (SGNPs) with sizes ranging from 16 up to 20 nm, entrapment efficiency 96%, and loading capacity 38.69%. The in vivo study indicated that the obtained nano-formulation of SIL boosts its anti-fibrotic effects. The SIL or SGNPs may induce their anti-fibrosis effects mainly through targeting and enhancing the hepatic expression of the protective microRNAs; miR-22, miR-29c, and miR-219a which resulting in suppressed expression of the main fibrosis mediators; TGFβR1, COL3A1, and TGFβR2, respectively. These effects together with the TGF-β1 lowering and Nrf2 stimulation resulted in blocking the pathological pathways that participated in the development of liver fibrosis including, TGF-β1/SMAD signaling, chronic inflammation, oxidative stress, and collagen deposition.

## Figures and Tables

**Table 1:** The primer sequences for predict target genes used in the study.

Gene	Sequence	
COL3A1	Forward	5′-AAC GGA GCT CCT GGC CCC AT-3′
	Reverse	5′-ATT GCC TCG AGC ACC TGC GG-3′
TGFβR1	Forward	5′-GCT GAC ATC TAT GCA ATG GG-3′
	Reverse	5′-ATA TTT GGC CTT AAC TTC TGT TC-3′
TGFβR2	Forward	5′-CCA GGG CAT CCA GAT CGT GTG-3′
	Reverse	5′-TAG TGT TCA GGG AGC CGT CTT-3′
GAPDH	Forward	5′-GGG TGT GAA CCA CGA GAA ATA-3′
	Reverse	5′-AGT TGT CAT GGA TGA CCT T-3′

**Table 2:** Effect of silymarin concentration on drug entrapment efficiency and loading capacity.

	Silymarin Concentration		
Parameters	0.482 mg/mL	1 mg/mL	1.2 mg/mL
Entrapment efficiency	90%	95%	96%
Drug loading capacity	19%	34%	38.69%

**Table 3:** The body weight gain, liver weight, and liver index of control rats and CCl_4_-induced fibrotic rats untreated or treated with SIL, GNPs, and SGNPs.

Groups		Parameters	
	Body Weight Gain (g)	Liver Weight (g)	Liver Index (%)
Control	101.50 ^a^ ± 11.24	9.08 ^c^ ± 0.64	2.59 ^d^ ± 0.21
CCl_4_	−44.14 ^c^ ± 10.07	13.22 ^a^ ± 0.71	6.41 ^a^ ± 0.26
CCl_4_+SIL	52.83 ^b^ ± 5.49	11.25 ^b^ ± 0.23	3.70 ^c^ ± 0.11
CCl_4_+GNPs	−30 ^c^ ± 9.92	11.50 ^b^ ± 0.48	5.16 ^b^ ± 0.32
CCl_4_+SGNPs	93.67 ^a^ ± 5.47	9.24 ^c^ ± 0.52	2.68 ^d^ ± 0.11

Data are expressed as mean ± SD (*n* = 6). Means of the groups in the same column with the same superscript letter are not significantly different while the means of the groups with different superscript letter are significantly differed (*p* ≤ 0.05) by ANOVA test using Post Hoc Test (Tukey).

**Table 4:** Effects of SIL, GNPs, and SGNPs on serum liver function parameters.

Groups	Parameters				
	AST (U/L)	ALT (U/L)	ALP (U/L)	Total Bilirubin (mg/dL)	Albumin (g/dL)
Control	111.5 ^d^ ± 12.44	43.33 ^d^ ± 7.42	101.3 ^d^ ± 13.79	0.40 ^c^ ± 0.10	4.11 ^a^ ± 0.21
CCl4	195.5 ^a^ ± 17.76	130.8 ^a^ ± 13.70	252.2 ^a^ ± 19.62	1.15 ^a^ ± 0.30	3.14 ^c^ ± 0.13
CCl4+SIL	134.3 ^bcd^ ± 12.75	61.17 ^c^ ± 9.02	148.7 ^c^ ± 16.72	0.75 ^b^ ± 0.21	3.49 ^bc^ ± 0.24
CCl4+GNPs	159.0 ^b^ ± 15.10	104.7 ^b^ ± 8.91	189.0 ^b^ ± 16.42	0.83 ^b^ ± 0.12	3.27 ^bc^ ± 0.27
CCl4+SGNPs	125.0 ^cd^ ± 10.14	51.8 ^cd^ ± 6.77	133.5 ^c^ ± 12.58	0.67 ^bc^ ± 0.10	3.60 ^b^ ± 0.08

Data are expressed as mean ± SD (*n* = 6). Means of the groups in the same column with the same superscript letter are not significantly differedent while the means of the groups with different superscript letter are significantly differed (*p* ≤ 0.05) by ANOVA test using Post Hoc Test (Tukey). AST, aspartate transferase; ALT, alanine transaminase; ALP, alkaline phosphatase.

**Table 5:** Therapeutic effects of SIL, GNPs, and SGNPs on redox parameters and total TGF-1β levels in hepatic tissue homogenate.

Groups	Parameters		
	MDA (nmol/g Tissues)	NRF2 (pg/mg Protein)	TGF-β1 (ng/mg Protein)
Control	11.68 ^c^ ± 2.46	109.4 ^a^ ± 9.48	7.24 ^d^ ± 2.04
CCl4	25.75 ^a^ ± 4.43	56.97 ^c^ ± 8.31	28.44 ^a^ ± 3.04
CCl4+SIL	17.30 ^b^ ± 2.13	68.88 ^bc^ ± 11.77	19.12 ^c^ ± 1.91
CCl4+GNPs	18.11 ^b^ ± 3.81	59.27 ^bc^ ± 7.60	23.40 ^b^ ± 2.53
CCl4+SGNPs	14.48 ^bc^ ± 2.06	76.30 ^b^ ± 14.95	18.05 ^c^ ± 1.97

Data are expressed as mean ± SD (*n* = 6). Means in the same column with common superscript letters are not significant and means with different superscript letters are significant (*p* ≤ 0.05) by ANOVA test using Post Hoc Test (Tukey). MDA, malondialdehyde; NRF2, nuclear factor-erythroid 2-related factor 2; TGF-β, transforming growth factor.

## Data Availability

Data will be available by request to the corresponding authors.
